# Risk factors for breakthrough vitreous hemorrhage after intravitreal anti-VEGF injection in age-related macular degeneration with submacular hemorrhage

**DOI:** 10.1038/s41598-018-28938-1

**Published:** 2018-07-12

**Authors:** Yong-Il Shin, Jae-Yun Sung, Min Sagong, Young-Hoon Lee, Young-Joon Jo, Jung-Yeul Kim

**Affiliations:** 1 Department of Ophthalmology, Chungnam National University College of Medicine, Daejeon, Republic of Korea; 2 Department of Ophthalmology, Yeungnam University College of Medicine, Daegu, Republic of Korea; 3 Department of Ophthalmology, Konyang University College of Medicine, Daejeon, Republic of Korea

## Abstract

To investigate the risk factors for breakthrough vitreous hemorrhage (VH) after intravitreal anti-vascular endothelial growth factor (anti-VEGF) injection in age-related macular degeneration (AMD) accompanied by submacular hemorrhage (SMH). We retrospectively reviewed the medical records of patients diagnosed with AMD combined with SMH, and enrolled 31 patients. We formed an age- and sex-matched control group of patients with submacular hemorrhage who did not develop breakthrough VH after intravitreal injection during 6 month follow-up. The mean patient age was 70.8 ± 10.3 years in the breakthrough VH group. Of the 31 patients, 8 were diagnosed with choroidal neovascularization (CNV), 22 with polypoidal choroidal vasculopathy (PCV), and 1 with retinal angiomatous proliferation (RAP). PCV was associated with a significantly higher incidence of VH (odds ratio, 35.01; *p* = 0.001). The size of the SMH was 22.7 ± 12.4 disc areas (DAs) in the breakthrough VH group and 5.4 ± 6.9 DAs in the control group, and was thus significantly related to the development of VH (*p* < 0.001). The risk of VH was significantly higher in those taking anticoagulants (*p* = 0.014). There was no significant difference between the types of anti-VEGF agents. When taking anticoagulant medications, a SMH of large diameter, and PCV subtype were risk factors for breakthrough VH after anti-VEGF injection.

## Introduction

Submacular hemorrhage (SMH) occurs when blood accumulates between the neurosensory retina and the retinal pigment epithelium of the macula, and is most often associated with age-related macular degeneration (AMD). SMH can also be caused by retinal arterial macroaneurysm, pathological myopia, trauma, and tumors^1,2^ and can causes sudden visual loss. Iron and hemosiderin^3^ are toxic to the retina and fibrin clots may tear the photoreceptor layer^4^. Blood clots serve as barriers between the retina and the choroid, inhibiting transfer of the oxygen and nutrients. Subretinal fibrosis and disciform scar changes^5–7^ may lead to serious visual impairment; appropriate treatment is essential.

The treatment options include observation if the amount of SMH is small; intravitreal injections of gas or tissue plasminogen activator (t-PA); pars plana vitrectomy with or without subretinal t-PA injections; photodynamic therapy (PDT); and intravitreal injection of anti-vascular endothelial growth factor (anti-VEGF)^1,8^. The complications and visual prognosis of each treatment differ by disease etiology, the extent of SMH, and the time that elapses prior to treatment. Also, visual recovery may be limited due to disease progression after treatment. Hence, there is no gold standard treatment for SMH.

Intravitreal anti-VEGF injection is a safe and effective treatment for AMD and is now widely used, and is associated with visual and anatomical improvements. Such treatment is less invasive than vitrectomy and can be performed without face-down position, which is essential for intravitreal gas injection for pneumatic displacement. In addition, there have been several reports of good visual outcomes in AMD patients with SMH after monotherapy with intravitreal anti-VEGF injection^9–12^.

However, complications such as subconjunctival hemorrhage, elevated intraocular pressure, endophthalmitis, cataract, and vitreous hemorrhage (VH) may develop after intravitreal injection^13,14^. Sometimes the VH is become naturally absorbed, and can be simply observed. However, vitrectomy may be necessary if a large dense VH develops. Therefore, our purpose was to define risk factors for VH developing after intravitreal anti-VEGF injection in AMD patients with SMH. In addition, we compared the prognosis of the VH and a control group after intravitreal anti-VEGF injection.

## Methods

### Study design

This study was a retrospective, case control study. We reviewed the medical records of patients receiving intravitreal anti-VEGF injections to treat AMD who were referred to the retina clinic of Chungnam National University Hospital from January 2008 to January 2017. The study protocol was approved by the Institutional Review Board of Chungnam National University Hospital. The requirement for obtaining informed patient consent was waived due to the retrospective nature of the study. The study adhered to the tenets of the Declaration of Helsinki.

### Participants

The inclusion criteria were as follows: 1) age ≥55 years; 2) an initial diagnosis of AMD treated via intravitreal anti-VEGF injection; 3) an SMH involving the central fovea at the time of diagnosis; 4) a hemorrhage size greater than one optic disc area (DA); 5) a thick hemorrhage, associated with bulging of the macular center; and 6) follow-up >6 months.

The exclusion criteria were: 1) any prior history of intravitreal injection or PDT; 2) any other intraocular disease; and, 3) any previous history of intraocular surgery except cataract surgery.

AMD with SMH included a total of 337 patients suitable for inclusion criteria. VH occurred in 31 patients after intravitreal injection during 6-month follow-up, and 87 of 306 patients were matched with the control group.

Multimodal images of fundus photography, fluorescein angiography (FA)/indocyanine green angiography (ICGA), and spectral-domain optical coherence tomography (SD-OCT) were used to diagnose AMD. Patients with branching vascular networks and distal nodular lesions evident on ICGA were diagnosed with polypoidal choroidal vasculopathy (PCV)^15^. Patients with characteristic retinal-retinal or retinal-choroidal anastomoses were diagnosed with retinal angiomatous proliferation (RAP)^16^. All other cases were diagnosed with choroidal neovascularization (CNV).

### Study procedure

We investigated whether VH occurred after intravitreal anti-VEGF injection in AMD patients with SMH during 6-month follow-up. Patients with VH and age- and sex-matched patients without VH constituted the study (Group 1) and control groups (Group 2). We compared the medical and surgical histories, anticoagulant medication use, visual acuity, AMD subtype, the type of anti-VEGF agent, and SMH size between the two groups.

Medical records were reviewed for the following data: age, sex, systemic disease, anticoagulant medication, duration of symptom, follow-up duration, best-corrected visual acuity (BCVA), slit-lamp examination, dilated fundus examination, SD-OCT (Cirrus HD-OCT, Carl Zeiss Meditec, Dublin, CA, USA) and FA/ICGA (HRA-2; Heidelberg Engineering, Dossenheim, Germany) findings.

According to medical records, all patients received intravitreal anti-VEGF injection via a 30-gauge needle to inject 0.05 mL through the pars plana.

The SMH size was recorded as a multiple of the optic DA of the affected patient, as revealed by fundus photography using Image J software. Data were interpreted by two graders (YIS and JYS) who were masked to subject characteristics. The average value of the results of the two examiners was used. The intergrader and intragrader agreements were ĸ = 0.911 and 0.943, respectively, indicating good reliability.

### Statistical analysis

Statistical analysis were performed using SPSS statistical software, version 22.0 (SPSS, Chicago, IL, USA). For statistical analysis, BCVA values were transformed to the logarithms of the minimum angle of resolution (logMAR). In the current study, the visual acuities of counting fingers and hand motion were considered to be 2.0 and 3.0 logMAR, respectively^17^. We used the chi-squared test, Fisher’s exact test, and the Mann–Whitney U-test, as appropriate, and multivariate logistic regression was performed to determine significance, odds ratios for developing vitreous hemorrhage, 95% confidence intervals (CIs) based on risk factors; anticoagulation medication, subtype of AMD, duration of symptoms, and extent of hemorrhage. A *p*-value < 0.05 was considered statistically significant.

### Data availability

Data supporting the findings of the current study are available from the corresponding author on reasonable request.

## Results

VH developed in 31 patients (31 eyes) (Group 1) who had received intravitreal anti-VEGF injection after diagnosis of AMD with SMH during the 6-month follow-up. Twenty-nine patients had vitreous hemorrhage after a single injection, and two patients had it after a second injection. The control group (Group 2) included 87 patients (87 eyes) who did not develop VH after intravitreal anti-VEGF injection after diagnosis of AMD with SMH during the 6-month follow-up; these patients were age-and sex-matched to Group 1 patients. There was no VH in the control group within 6 months after injection.

In Group 1, vitrectomy was performed on 11 eyes (35.5%) because the VH was not spontaneously absorbed. We found no significant difference in age, sex, intraocular pressure, laterality, lens status, or medical history (HTN and DM) between the two groups. The follow-up duration of the two groups did not differ significantly (Group 1, 18.2 ± 16.6 months, Group 2, 20.3 ± 19.1 months; *p* = 0.632). However, the incidence of VH was significantly higher in patients taking anticoagulants (*p* = 0.003) (Table 1).

**Table 1:** Baseline data of patients with submacular hemorrhage receiving intravitreal anti-VEGF injection.

	Group 1 (n = 31); breakthrough VH(+)	Group 2 (n = 87); breakthrough VH (−)	*p-*value
Age (years)	70.8 ± 10.3	70.9 ± 10.3	0.958^†^
Sex (M/F)	21/10	60/27	0.900^*^
Intraocular pressure (mmHg)	15.3 ± 3.3	14.6 ± 3.8	0.475^†^
Laterality (OD/OS)	10/21	40/47	0.184^*^
Lens status (phakic/pseudophakic)	25/6	66/21	0.586^*^
Hypertension (no. (%))	16(51.6%)	43(49.4%)	0.834^*^
Diabetes mellitus (no. (%))	5(16.1%)	17(19.5%)	0.675^*^
Anticoagulant medication (no. (%))	14(45.2%)	16(18.4%)	0.003^*^
Follow up duration (months)	18.2 ± 16.6	20.3 ± 19.1	0.632^†^

VEGF (vascular endothelial growth factor), VH (vitreous hemorrhage).

^*^Chi-square test, ^†^Mann-Whitney U test.

Of the 31 eyes that developed VH, CNV was diagnosed in 8 eyes, PCV in 22 eyes, and RAP in 1 eye. The AMD PCV subtype was significantly more prevalent in Group 1 (*p* = 0.004). Symptom duration was 17.2 ± 23.4 days in Group 1 and 46.1 ± 65.4 days in Group 2, thus significantly shorter in the VH group (*p* = 0.004).

The SMH size was 22.7 ± 12.4 DAs in the VH group and 5.4 ± 6.9 DAs in the control group, thus significantly larger in the VH group (*p* < 0.001). The type of anti-VEGF agent used did not influence the VH risk.

The initial BCVA was 1.21 ± 0.71 in Group 1 and 0.77 ± 0.54 in Group 2, thus significantly poorer in the VH group (*p* = 0.022). However, the final BCVA after treatment were 1.31 ± 0.88 and 0.90 ± 0.80, respectively, in the two groups, thus slightly poorer in the VH group. However, there were no significant differences (Table 2).

**Table 2:** Clinical characteristics of patients with submacular hemorrhage and outcomes after anti-VEGF injection.

	Group 1 (n = 31); breakthrough VH(+)	Group 2 (n = 87); breakthrough VH (−)	*p-*value
Subtype of AMD (no. (%))			0.004^†^
CNV	8(25.8%)	46(52.9%)	
PCV	22(71.0%)	32(36.8%)	
RAP	1(3.2%)	9(10.3%)	
Duration of symptoms (days)	17.2 ± 23.4	46.1 ± 65.4	0.004^‡^
Extent of hemorrhage (disc areas)	22.7 ± 12.4	5.4 ± 6.9	<0.001*
0–10	4	71	
11–20	11	8	
>20	16	8	
Type of anti-VEGF agent (no. (%))			0.065*
Bevacizumab	15(48.4%)	23(26.4%)	
Ranibizumab	12(38.7%)	42(48.3%)	
Aflibercept	4(12.9%)	22(25.3%)	
Visual acuity (logMAR)			
Initial	1.21 ± 0.71	0.77 ± 0.54	0.022^‡^
Final	1.31 ± 0.88	0.90 ± 0.80	0.571^‡^

VEGF (vascular endothelial growth factor), VH (vitreous hemorrhage), AMD (age-related macular degeneration), CNV (choroidal neovascularization), PCV (polypoidal choroidal vasculopathy), RAP (retinal angiomatous proliferation).

^*^Chi-square test, ^†^Fisher’s exact test, ^‡^Mann-Whitney U test.

**Figure 1:**
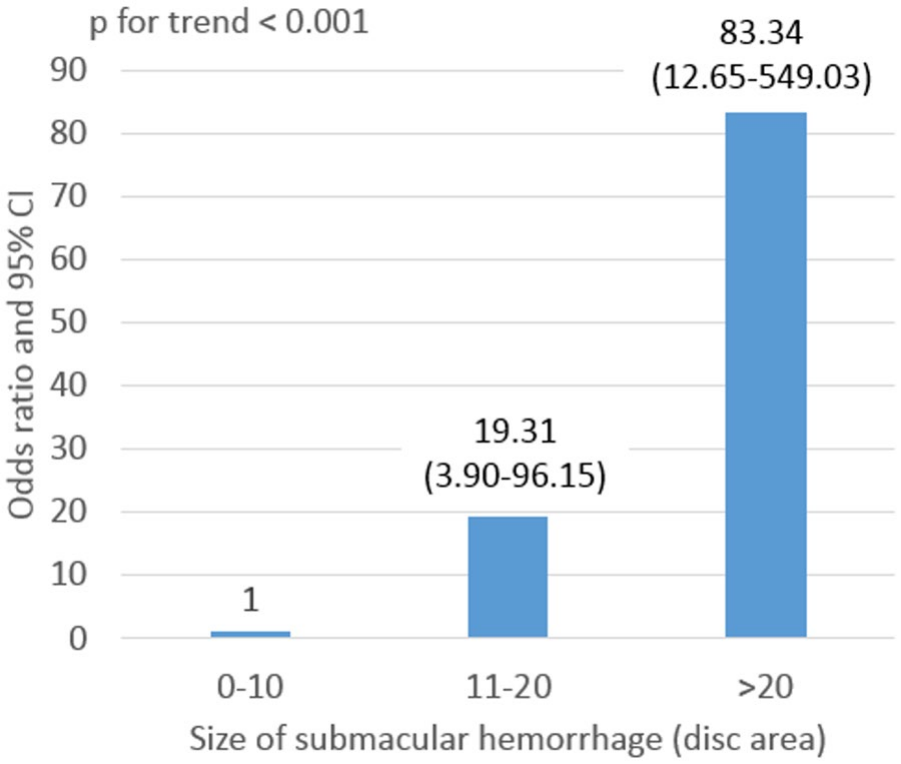
Odds ratios for, and 95% confidence intervals of, the risk of vitreous hemorrhage after intravitreal anti-vascular endothelial growth factor injection by submacular hemorrhage size.

Multivariate logistic regression showed that patients taking anticoagulants were significantly more likely to develop VH (odds ratio [OR], 5.86; 95% CI, 1.43–24.09; *p* = 0.014). Patients with PCV were also significantly more likely to develop VH (OR, 35.01; 95% CI, 4.48–273.63; *p* = 0.001). A larger hemorrhage size was strongly associated with a significantly greater risk of VH (*p* for trend < 0.001). Patients with hemorrhages 11–20 DAs in size were 19.31-fold more likely to develop VH than those with hemorrhages 0–10 DAs in size (95% CI, 3.90–96.15; *p* < 0.001). Those with hemorrhages >20 DAs in size were 83.34-fold more likely to develop VH than those with hemorrhages 0–10 DAs in size (95% CI, 12.65–549.03; *p* < 0.001) (Fig. 1). We found no association between VH development and other possible risk factors (RAP or symptom duration). Table 3 shows the results of analysis of possible predisposing factors for VH after intravitreal anti-VEGF injection of AMD patients with SMH.

**Table 3:** Analysis of possible predisposing factors for vitreous hemorrhage after intravitreal anti-VEGF injection of AMD with submacular hemorrhage.

Risk factors	Multivariate-adjusted		p*
	Odds ratio	95% CI	
Anticoagulation medication	5.86	1.43–24.09	0.014
Subtype of AMD			
CNV	1		
PCV	35.01	4.48–273.63	0.001
RAP	22.87	0.98–534.50	0.052
Duration of symptoms	0.99	0.96–1.02	0.454
Extent of hemorrhage (disc areas)			
0–10	1		
11–20	19.31	3.90–96.15	<0.001
>20	83.34	12.65–549.03	<0.001

VEGF (vascular endothelial growth factor), AMD (age-related macular degeneration), CNV (choroidal neovascularization), PCV (polypoidal choroidal vasculopathy), RAP (retinal angiomatous proliferation).

*Multivariate logistic regression analysis.

Treatment was well-tolerated and there were no cases of infectious endophthalmitis or retinal pigment epithelial tear. No severe ocular or systemic adverse event was noted.

## Discussion

Anti-VEGF treatments for AMD are widely used today. The MARINA and ANCHOR trials proved that such treatment was effective. However, these studies excluded large numbers of predominantly hemorrhagic CNV patients^18,19^. Therefore, no established treatment for SMH associated with AMD is yet available. Vitrectomy, t-PA injection, and intravitreal gas injection have been performed singly or in combination to remove or transfer SMHs. However, subretinal manipulations such as retinotomy or t-PA injection into the subretinal area are associated with various complications. Also, intravitreal gas injection is associated with risks of rebleeding, VH, and elevation of intraocular pressure. In addition, such injections are difficult to perform when a patient is in poor general condition and cannot lie prone^1,8^.

In the present era of antiangiogenic agents, VEGF inhibitors may be a useful for alternative initial treatments for SMH. Recently, several reports have indicated that anti-VEGF therapy alone improved visual acuity in patients in AMD with SMH. Chang *et al*.^9^ reported the first prospective study of anti-VEGF injection in such patients. Ranibizumab monotherapy was delivered to seven patients. Three patients exhibited VA improvements of more than two lines during the 12-month follow-up period (43%). No patient experienced a decrease in VA of more than two lines. Cho *et al*.^10^ studied 27 PCV patients with SMH and reported that 10 eyes (37%) exhibited VA improvements of ≥0.3 logMAR, 11 eyes (40.7%) stabilized, and six (22.2%) exhibited VA decreases ≥0.3 logMAR 12 months after anti-VEGF injection. Although the sample sizes were small, all studies found that intravitreal injections alone improved visual acuity without any serious side effects. However, the classifications and the degree of SMH were different, so further studies are required.

Spontaneous VH is rare in AMD cases^20^. Most such cases are preceded by SMH^21^. Patients with massive SMH may be treated via pneumatic displacement or t-PA injection, which may cause VH^22–24^. Hesse *et al*.^24^ reported that higher t-PA doses tended to increase the VH risk. Wu *et al*.^25^ reported 18 cases (15%) of breakthrough VH after intravitreal t-PA injection and pneumatic displacement in 120 SMH patients. Of these, six underwent vitrectomy. The incidence of VH following intravitreal injection of agents treating SMH varies. Cho *et al*.^9^ reported that three of 27 eyes (11.1%) underwent vitrectomy to treat VH.

The risk factors for massive SMH^26^ or developing massive SMH after inttravitreal injections^27^ have been reported in AMD patients. There has been a study of risk factors for VH after t-PA in patients with AMD accompanied by SMH^25^. However, our study was a new and important study because there have been no reports of risk factors for VH after intravitreal injection in patients with AMD accompanied by SMH.

We evaluated the risk factors for breakthrough VH after intravitreal anti-VEGF injection of patients with AMD. We found that the baseline demographic data (laterality, lens status, HTN, and DM) did not differ significantly between the two groups. However, unlike what was reported by Hasegawa *et al*.^20^ and Wu *et al*.^25^, we found that more VH patients (Group 1) took anticoagulants. Such medications affect the hemorrhagic complications in AMD patients^28–30^. Tilanus *et al*.^26^ reported that VH occurred more frequently in patients on anticoagulants (OR: 11.6; 95% CI, 3.1–44.3), consistent with our findings (OR, 5.86; 95% CI, 1.43–24.09). Older AMD patients are more likely to have HTN, and ischemic heart and cerebrovascular disease, and are often on anticoagulants or antiplatelet agents. Therefore, detailed history-taking is required to explore the future risks of severe SMH or VH.

We evaluated the SMH etiologies of the two groups and their risks of VH. Of 31 eyes exhibiting VH, 22 (71.0%) had PCV; this was of statistical significance. PCV patients were significantly more likely to develop VH after intravitreal injection (OR, 35.01; 95% CI, 4.48–273.63; *p* = 0.001).

PCV is associated with a higher incidence of hemorrhagic complications, such as SMH, hemorrhagic pigmented epithelial detachment, and VH, than other types of AMD^31,32^. In addition, the vascular abnormalities of typical PCV may be susceptible to pressure gradients caused by intravitreal injection. Therefore, SMH secondary to PCV is more likely to develop VH after intravitreal injection. In addition, the PCV prevalence is higher in Asians, and is more likely to be accompanied by SMH^33–35^. Some studies have reported the development of subretinal hemorrhage associated with complications after PDT and intravitreal anti-VEGF injections for PCV^36,37^. But there have been no reports on epidemiological and clinical features of VH after intravitreal anti-VEGF injections in patients with PCV with SMH.

The SMH size was 22.7 ± 12.4 DAs in the VH group and 5.4 ± 6.9 DAs in the control group, thus significantly larger in the VH group (*p* < 0.001). A larger hemorrhage was strongly associated with a significantly greater VH risk (*p* for trend < 0.001); those with hemorrhages >20 DAs in size were 83.34-fold more likely to develop VH compared with those with hemorrhages 0–10 DAs in size (95% CI, 12.65–549.03; *p* < 0.001) (Fig. 1), consistent with the results of other studies^25,38^.

The types of anti-VEGF agent used did not differ between the two groups. The initial BCVA was significantly poorer in the VH group (1.21 vs. 0.77; *p* = 0.022), perhaps because the SMHs were larger. Also, the BCVA at final follow-up was somewhat poorer in Group 1, but there was no significant difference between the two groups (1.31 vs. 0.90; *p* = 0.571).

Our study had some limitations. This was a retrospective study with relatively small patient numbers. Further prospective studies with more patients are required. SMH was sized based on the DAs of individual patients. Thus, accurate measurement was difficult; the data may be affected by disc size. However, direct size measurement is associated with refractive error and errors in camera magnification; our measurement method may in fact appropriately determine the extent of SMH. Finally, the effect of hemorrhage thickness should be further investigated.

In conclusion, in AMD patients with SMHs, the use of anticoagulant medication, large-diameter hemorrhage, and disease of the PCV subtype, were risk factors for breakthrough VH after anti-VEGF injection. Ophthalmologists should know the risk factors and explain the risk of VH after intravitreal injection to all patients.
